# Optically Isotropic, Colorless, and Flexible PITEs/TiO_2_ and ZrO_2_ Hybrid Films with Tunable Refractive Index, Abbe Number, and Memory Properties

**DOI:** 10.1038/s41598-017-08544-3

**Published:** 2017-08-11

**Authors:** Tzu-Tien Huang, Shun-Wen Cheng, Chia-Liang Tsai, Guey-Sheng Liou

**Affiliations:** Functional Polymeric Materials Laboratory, Institute of Polymer Science and Engineering, National Taiwan University, 1 Roosevelt Road, 4th Sec., Taipei, 10617 Taiwan

## Abstract

A series of novel polyimidothioethers (PITEs) and the respective polymer hybrids of titania or zirconia with fantastic thermal stability and optical properties have been successfully prepared. These colorless PITEs with high transparency were synthesized by Michael polyaddition from commercially available dithiol and bismaleimides monomers. The PITE with sulfide and hydroxyl groups (S-OH) and the corresponding hybrid films declare ultra-lowest birefringence value of 0.002 and tunable refractive index (1.65–1.81 for S-OH/titania and 1.65–1.80 for S-OH/zirconia), implying large potential to the optical applications in the future. Moreover, the S-OH/zirconia hybrid films exhibit higher Abbe’s number and optical transparency than those of S-OH/titania system because larger energy band gap of ZrO_2_. Furthermore, by adding titania and zirconia as electron acceptor into S-OH system, the charge transfer complex can be facilitated and stabilized caused by the lower LUMO energy level of hybrid materials. Consequently, the devices of memory prepared from these polymer films of hybrid showed interesting and adjustable memory behavior from DRAM, SRAM, to WORM at various titania or zirconia contents with a large ON/OFF ratio (10^8^), denoting that the memory devices derived from these highly transparent novel S-OH/TiO_2_ and S-OH/ZrO_2_ hybrid films are attractive for the electrical applications.

## Introduction

Polymer-inorganic hybrid materials associate plentiful dominances of organic polymers and inorganic materials, such as outstanding optical, electrical and mechanical properties, thermal stability, and processability, thus, draw a lot of attention in optoelectronic applications^1–5^. There are some valuable reports related to the basic methodology for developing polymers with high refractive index (RI; n) and other important parameters, such as Abbe’s number (Vd) and birefringence (∆n) for the applications of anti-refractive coatings, micro-lenses for CMOS image sensors, and LEDs encapsulants^6–10^.

It is well known that the highly transparent thermoplastic polyimidothioethers (PITEs) prepared from bismaleimides (BMIs) via Michael polyaddition exhibited attractive optical and thermal characteristics, therefore making them greatly suitable for advanced composites and electronics, and also have positive value to be used in electrical applications^11^. Moreover, introduction of the sulfur-containing moieties into polymers could enhance RI which is demanding for the optical applications^12–18^.

In the previous studies^19–25^, the polyimide (PI)/titania hybrids revealed higher RI and thermal stability by increasing TiO_2_ content. However, the transparency and Vd of PI/titania hybrids deteriorated severely at 400 nm when TiO_2_ content increased. Therefore, the selection of inorganic materials in the system of hybrid for enhancing the Vd and RI without sacrificing the optical transparency in the visible light area still is a crucial change. Recently, our group incorporated ZrO_2_ into the PI matrices^26^, and demonstrated that the obtained PI/zirconia films exhibited higher Vd and optical transparency in the visible light region because that the energy band gap of ZrO_2_ (5.0–5.85 eV) is much larger than that of TiO_2_ (3.2 eV)^26, 27^.

Controlling over distribution and particle size to achieve homogeneous dispersion of the inorganic building blocks within nanoscale domain size in the organic matrix is an important and challenging issue for obtaining hybrid materials with both high RI and transparency. Among the previous studies, chemical reaction based on *in-situ* sol-gel hybridization approach would resolve the agglomeration issue of nanoparticles by controlling inorganic/organic interfacial interactions at different molecular and nanometer length scales^19, 26^. On the contrary, materials with extremely low RI which are defined as antireflective (AR) coating materials could also be achieve by surface sol-gel methods and self-masking, one-step process and they attract much attention for great application potential and good development prospective^28–31^.

Lately, the hybrids of polymer have also attracted attention in the memory devices application, because the incorporation of ancillary components as donors or acceptors of electron into the polymers could enhance the charge transfer (CT) complex formation^27, 32^. In our previous studies, by introducing inorganic materials TiO_2_^23^ or PCBM^33^ as the strong acceptor of electron into the PI system, the obtained memory devices manifested attractive behaviors of memory because of the lower LUMO energy level that would facilitate and stabilize the CT, resulting in tunable memory retention time. In addition, these PI hybrid films of TiO_2_ or ZrO_2_ could also avoid the disability of decreasing ON/OFF ratio at higher TiO_2_ or ZrO_2_ contents at the hands of the depressed conductivity in the OFF state. Mechanism of memory behavior for these hybrids of polymer can be ascribed to the transfer of charge; thus, the homogeneous dispersion of inorganic particle with nanoscale domain size in polymer matrix is crucial for devices of memory. Herein, the highly transparent PITEs with pendant hydroxyl groups were prepared in this study; the memory behavior and optical properties such as transparency, Vd, and RI of the resulted flexible PITEs/TiO_2_ or ZrO_2_ hybrid films are investigated in this study.

## Results

### Polymer synthesis and characterization

**Figure 1:**
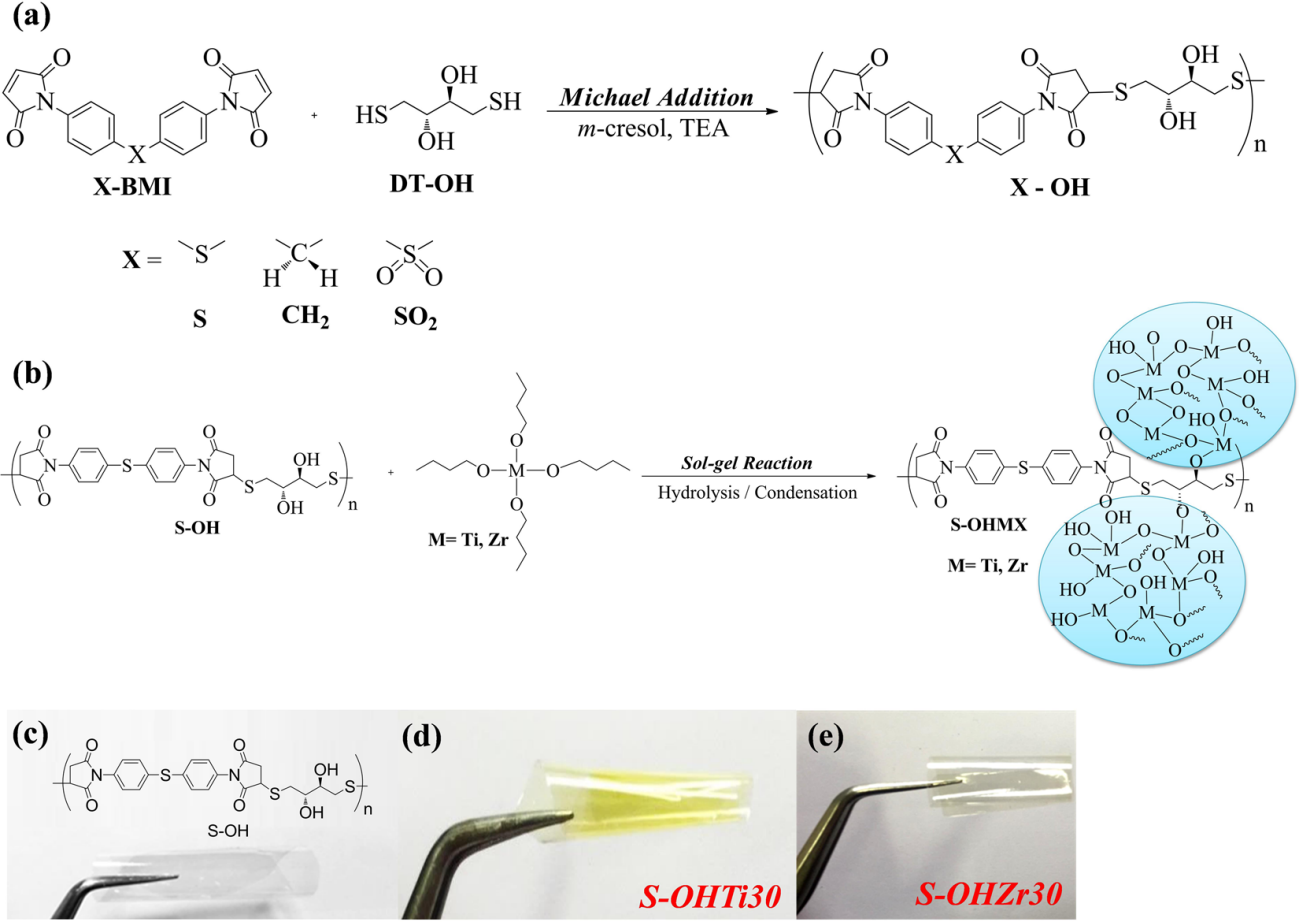
Representative flexible, transparent optical films and their structures. (**a**) Synthesis of Polyimidothioethers. (**b**) Synthesis and Structure of S-OH/TiO_2_ and S-OH/ZrO_2_ Hybrids. (**c**) Film of S-OH. (**d**) Film of S-OHTi30. (**e**) Film of S-OHZr30. (thickness: 20 ± 5 µm).

These PITEs could be readily obtained by the Michael polyaddition of commercially available dithiol DT–OH with bismaleimides, and the reaction was easily fulfilled with basic TEA as catalyst in m-cresol at 25 °C for 4 h (Fig. 1). The viscosity of polymer solution increased gradually during the procedure of polymerization without any volatile molecule evolution. Finally, the PITEs were precipitated in fiber-like form with white color and qualitative high yield (99.0%) when pouring the deriving polymer solution in methanol slowly. The sulfur-containing bismaleimide and dithiol used in this study exhibited higher RI and optical transparency among the obtained thermoplastic and colorless PITEs with excellent thermal characteristics. The inherent viscosity, solubility behavior, and the molecular weight of the synthesized PITEs are summarized in Table S1 (ESI†). These PITEs could dissolve in DMF, DMSO, DMAc, and NMP solvents and also could be casted as solution into transparent and flexible films as shown in Fig. 1c. FT-IR spectra of the PITEs are depicted in Figure S1 (ESI†). For S-OH, 3000 to 3750 cm^−1^ (O–H stretch), characteristic imide absorption bands at 1780 cm^−1^ (asymmetrical C = O), 1710 cm^−1^ (symmetrical C = O), 1383 cm^−1^ (C–N), 1082 cm^−1^ (Ar–S–Ar str.), and 734 cm^−1^ (imide ring deformation).

### Synthesis and characterization of PITE hybrids

The sol-gel reaction of S-OH with TiO_2_ or ZrO_2_ precursors was applied to prepare S-OH/TiO_2_ and S-OH/ZrO_2_ hybrid (S-OHMX) films depicted in Fig. 1, and the formations of the reaction are also summed up in Table 1. Figure 1 also shows that the hydroxyl groups in the backbone of S-OH would contribute reactions sites for the bonding between organic and inorganic materials, affording flexible, transparent, and homogeneous films of hybrid. In Figure S2 (ESI†), FT-IR spectra of S-OHTi50 and S-OHZr50 films of hybrid display broad absorption bands in the range of 2750 to 3700 cm^−1^ (O–H stretch) with larger intensity of signal than the pristine S-OH because of the new forming hydroxyl groups in the TiO_2_ and ZrO_2_. Moreover, the inorganic absorption bands of Ti–O–Ti and Zr-O-Zr at 650–800 and 600–650 cm^−1^, could be observed, respectively^19, 26^.

**Table 1:** Thermal properties of PITEs and S-OH hybrid films with TiO_2_ and ZrO_2_.

Index	Tg (°C)^a^	α before T_g_ (μm/m °C)^b^	Reactant composition /wt%		Hybrid film Inorganic content/wt%		T_d_^5^ (°C)^d^		T_d_^10^ (°C)^d^		R_w800_/%^*e*^
			Polymer	Ti(OBu)_4_/Zr(OBu)_4_	Theoretical	Experimental^*c*^	N_2_	Air	N_2_	Air	
S-OH	101	155	100	0	0	0	280	290	290	300	27.3
CH_2_-OH	109	65.8	100	0	0	0	290	290	300	305	30.5
SO_2_-OH	113	118.4	100	0	0	0	260	265	280	285	30.5
S-OHTi10	120	105	67.8	32.2	10	10.5	285	295	300	300	28.4
S-OHTi30	145	86	35.4	64.6	30	30.4	310	300	360	340	50.2
S-OHTi50	160	82	19.0	81.0	50	46.6	315	310	365	365	55.4
S-OHZr10	115	100	74.3	25.7	10	10.4	295	270	330	305	17.5
S-OHZr30	135	85	42.8	57.2	30	30.9	305	300	340	335	54.3
S-OHZr50	172	65	24.3	75.7	50	44.6	310	305	345	340	59.0

^a^Glass transition temperature and CTE data were as determined TMA measurements.

^b^Experimental content of inorganic part measured from TGA curves in air.

^c^Temperature at which 5% and 10% weight loss, and Residual weight percentages under nitrogen flow at 800 °C were also measured by TGA.

### Thermal properties of PITEs, S-OH/TiO_2_ and S-OH/ZrO_2_

The thermal properties of the PITEs, S-OH/TiO_2_ and S-OH/ZrO_2_ hybrid films (S-OHMX) were measured by TMA and TGA, and the results are listed in Table 1. The thermoplastic PITEs with high thermal stability and large thermal process window between Tg and Td revealed the usefulness for injection molding processes as shown in Figure S3 (ESI†) and Figure S4 (ESI†). Furthermore, the Tg of S-OH hybrid films increased with increasing inorganic content, and could be promoted to 160 °C and 172 °C at 50 wt% TiO_2_ and 50 wt% ZrO_2_ content, respectively, as shown in Figure S3 (ESI†). Moreover, the values of an important reference parameter called coefficient of thermal expansion (CTE) for polymer films in the microelectronic application are also summarized in Table 1. Roughly, organic matrixes usually have much higher CTE value than that of inorganic reinforced components. Thus, the resulting hybrid materials could effectively suppress CTE when the volume fraction of inorganic nanoparticles increased.

### Optical properties of PITEs and hybrid films

**Figure 2:**
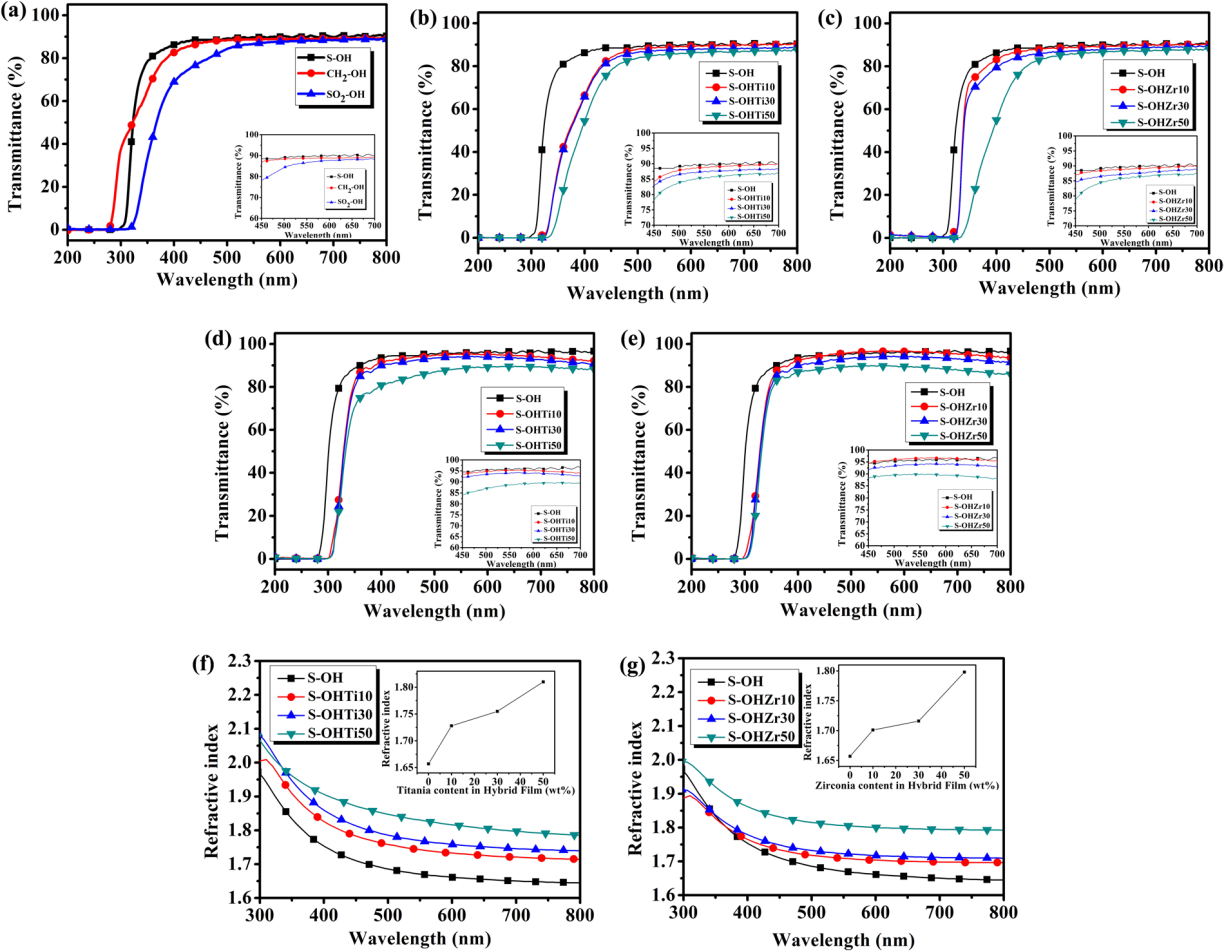
Measurements of optical properties. Optical transmission spectra of PITE, S-OHTiX and S-OHZrX hybrid films which are thick (**a**,**b**,**c**) (thickness: 20 ± 5 μm); and films which are thin (**d**,**e**) (thickness: 500–600 nm). Inset figures display the transmission spectra of hybrid films which are thin and thick in 450–700 nm of wavelength. Variation of the RI with wavelength for the hybrid films of (**f**) S-OHTiX and (**g**) S-OHZrX. Inset figures display the RI at 633 nm with diverse TiO_2_ and ZrO_2_ content.

The UV-vis transmission spectra of PITEs, S-OH/TiO_2_ and S-OH/ZrO_2_ hybrid thin and thick films with thickness of 500–600 nm and 20 ± 3 μm, respectively, were measured as summed up in Table 2 and Fig. 2. The transparent PITE film derived from the dithiol and sulfur-containing bismaleimide reveals enhanced RI. Thus, the transparency of S-OH thin film could reach 88% at 400 nm with RI of 1.657. Different from our previous research, these new PITEs films show remarkable results of optically isotropic feature with the ultra-lowest birefringence value of 0.002^11^ comparing to other polyimides with high RI. The most common optical materials such as glass is also isotropic and do not affect the polarization when the light passes through it. However, some materials which exhibit non-ignorable birefringence will change the polarization of light. There are many kinds of applications using optically isotropic materials, for example, liquid-crystal display (LCD). In this research, we use PITEs films to prepare resistive memory device which may have connection to optical applications in the future, denoting to the large potential in this kind of research. Furthermore, S-OH, exhibiting the highest RI and transparency among these three kinds of PITEs, was chosen as polymer matrix to prepare the S-OH/TiO_2_ and S-OH/ZrO_2_ hybrids. However, the optical transparency of the corresponding hybrid films of S-OH/TiO_2_ decreased apparently at 400 nm caused by the low energy band gap of TiO_2_ (3.2 eV), bringing about hybrid films with pale yellow color as shown in Fig. 1d even that the domain size TiO_2_ is smaller than 10 nm (Figure S5 (ESI†)). In addition, when the amount of inorganic part in the hybrid film increased, there would be a slight variation in the particle size that also could enhance the red-shift phenomenon^26^. Contrarily, the S-OH/ZrO_2_ hybrid films shown in Fig. 1e could keep higher transparency and colorless in the visible light due to the larger energy band gap of zirconium dioxide (5.0–5.85 eV) than the respective S-OH/TiO_2_ system,. The TEM image of hybrid material S-OHZr30 shown in Figure S6 (ESI†) depicts that the ZrO_2_ has good dispersion with size of domain smaller than 10 nm, resulting in lower cut-off wavelengths and fantastic optical transparency of these S-OH/ZrO_2_ hybrid films. The nanoparticles of hybrid system in the TEM pictures have been circled. In the following pictures, we can find that the particle sizes are in the range from 3 to 7 nm (the black dots); thus, the average particle size of 5 nm was defined in the statistical data curve. The RI diagrams in the range of 300–800 nm of these hybrid films with different TiO_2_ and ZrO_2_ content are summarized in Fig. 2, and the inset figures display the variety of RI at 633 nm. With amount of inorganic increasing, the RI enhanced, implying that the inorganic precursors of Ti–OH or Zr–OH groups could successfully form the structures of Ti–O–Ti and Zr–O–Zr by sol-gel reaction. Thus, greatly increase the RI. Moreover, Abbe number (Vd: variation of RI versus wavelength) is an decisive parameter for optical materials that indicates lower optical dispersion for the materials with high value of Vd. Attractively, S-OH/ZrO_2_ hybrid system not only could increase the RI effectively, but also upgrade Vd more efficiently than the respective TiO_2_ hybrid system. To determine the advanced values for optical applications, the S-OH/ZrO_2_ hybrid optical films with tunable RI effect have higher Vd and transparency than those of S-OH/TiO_2_ hybrid films. Merging the results of optical RI reaching to 1.80 at 633 nm and Vd of 36.8, film thickness and flexibility, the S-OHZr50 hybrid film exhibited the best optical transparency.

**Table 2:** Optical properties of S-OH and S-OH hybrid films with TiO_2_ and ZrO_2_.

	λ_0_ (nm)^*a*^	T_400_ (%)^*b*^	T_450_ (%)^*b*^	*n* ^*c*^	∆n^*d*^	*V*_d_^*e*^
S-OH	300/280	88/94	89/95	1.657	0.0020	16.23
S-OHTi10	314/296	67/92	84/94	1.728	0.0038	18.22
S-OHTi30	322/297	66/90	83/92	1.755	0.0063	18.88
S-OHTi50	326/300	54/81	78/84	1.810	0.0091	17.93
S-OHZr10	316/294	84/93	87/95	1.701	0.0034	31.73
S-OHZr30	322/295	80/90	85/92	1.716	0.0052	35.50
S-OHZr50	326/296	56/87	79/88	1.798	0.0087	36.81

^a^The cutoff wavelength (λ_0_) from the UV-vis spectra of polymer thick/thin films (thickness: 20 ± 3 μm/500–600 nm).

^b^Transmittance of polymer thick/thin films at 400 nm and 450 nm.

^c^RI at 633 nm. ^d^∆n = n_TE_ − n_TM_ using a prism coupler. ^e^Vd = n_587.56_ − 1/n_486.1_ − n_656.3_.

### Memory device characteristics and switching mechanism

UV-vis absorption spectra of PITEs are shown in Figure S7 (ESI†), and the energy band gap (Eg) could be estimated by onset wavelength of the optical absorption. Cyclic voltammetry (CV) was used to obtain the electrochemical properties of PITEs under nitrogen atmosphere employing 0.1 M tetrabutyl-ammonium perchlorate (TBAP) as the supporting electrolyte. The CV diagrams of PITEs are depicted in Figure S8 (ESI†), and energy levels of HOMO were calculated by onset oxidation. The redox potential of PITEs and the corresponding energy values of HOMO and LUMO were summarized in Table S2.

**Figure 3:**
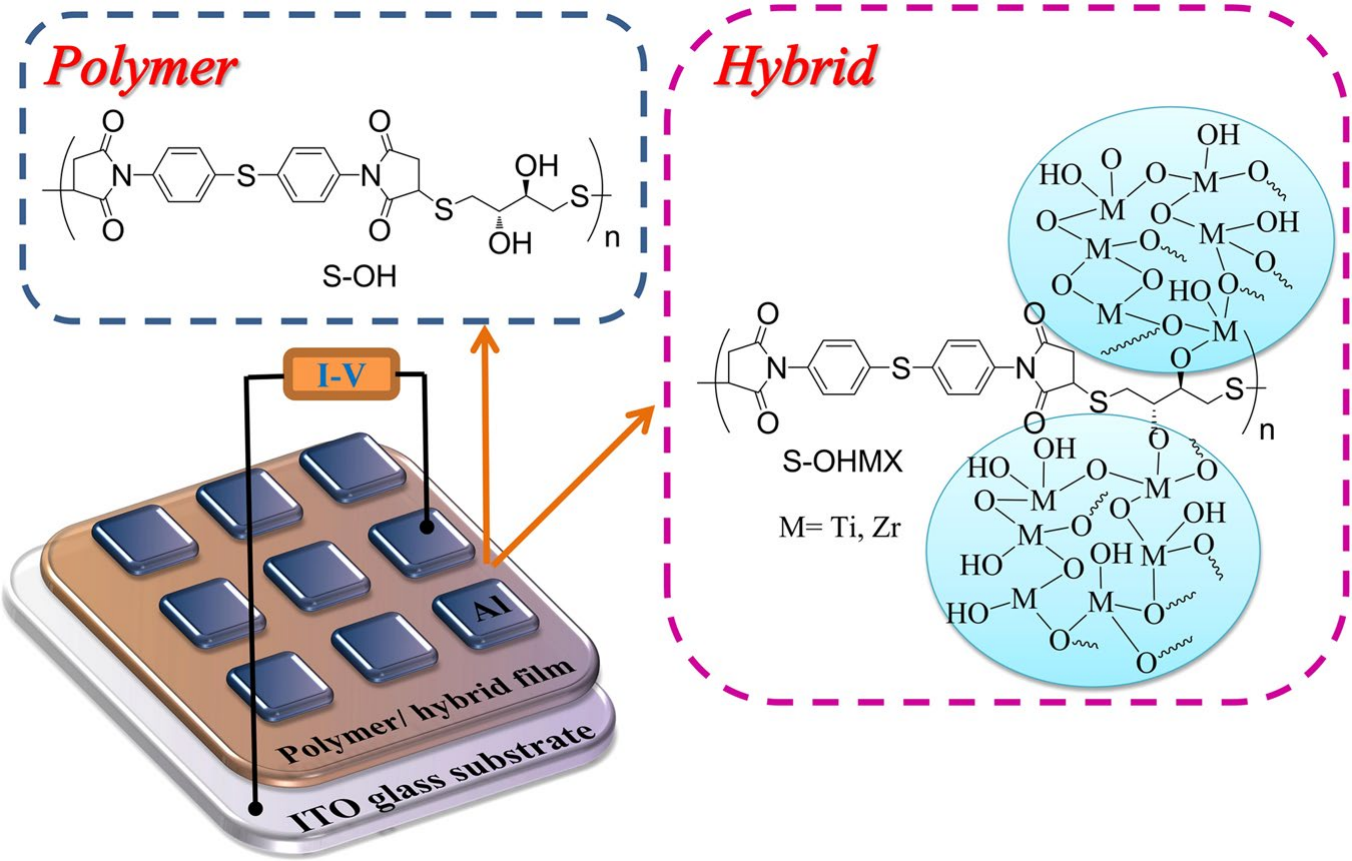
Chemical Structures of S-OH, S-OH/TiO_2_ or ZrO_2_, and the diagram of the memory device’s structure composing of a polymer or hybrid film sandwiched between aluminum top and ITO bottom Electrodes.

The memory behavior of S-OH was evaluated by current-voltage (I–V) curves during the potential sweep of an ITO/polymer/Aluminum sandwich-shaped device as shown in Fig. 3. Aluminum and ITO were acted as electrodes used to apply voltage. The thickness of polymer films were optimized about 50 nm due to the thickness effect on memory behavior which was mentioned before^34^. Figure S9 (ESI†) exhibits I-V curves of PITEs, and the devices only retained in OFF state with a current range 10^−13^ to 10^−15^ A without switching to the ON state both in negative and positive sweeps up to −6 V and 6 V, correspondingly, denoting non-memory characteristic. For better understanding the PITEs memory behavior, the simulation of a molecule of the basic unit with Gaussian 09 program was accomplished by DFT/B3LYP/6-31G(d). Those experimental values were in the affirmative with the LUMO and HOMO energy levels derived from the molecular simulation and isosurface charge density of the basic units is also summed up in Figure S10 (ESI†).

**Figure 4:**
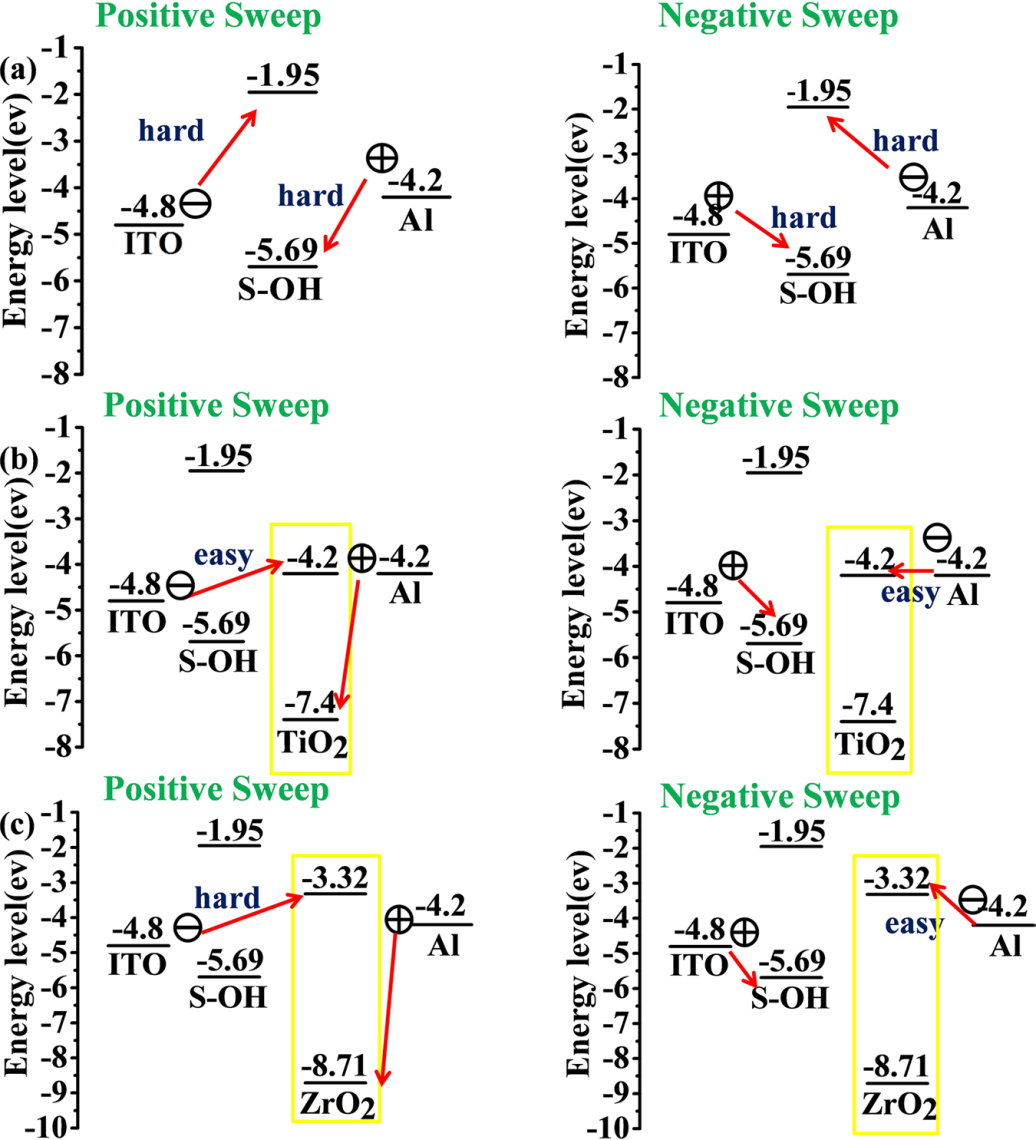
HOMO and LUMO energy levels along with the work function of the electrodes. (**a**) S-OH. (**b**) S-OH and TiO_2_. (**c**) S-OH and ZrO_2_.

According to the description of previous report^35^, the electrons at the LUMO (ON state) will be firstly transmitted from the HOMO when applied electric field compasses voltage of which causes accumulation of energy in HOMO, assembling a charge transfer complex by many kinds of ways that can switch the device to ON state. Interestingly, the structrue of S-OH has no capable donor group and acceptor group to perform the memory properties. Moreover, the memory device derived from S-OH behaved non-memory characteristic could be attributed to the imide ring in S-OH has the opposite direction between electron-withdrawing and electron-donating groups. Thus, the electron in LUMO is difficult to be stabilized due to the high energy band gap. Consequently, charge transfer could not steadily exist through multiple approaches to form the stable CT complexes which are more conductive as shown in Fig. 4a.

**Figure 5:**
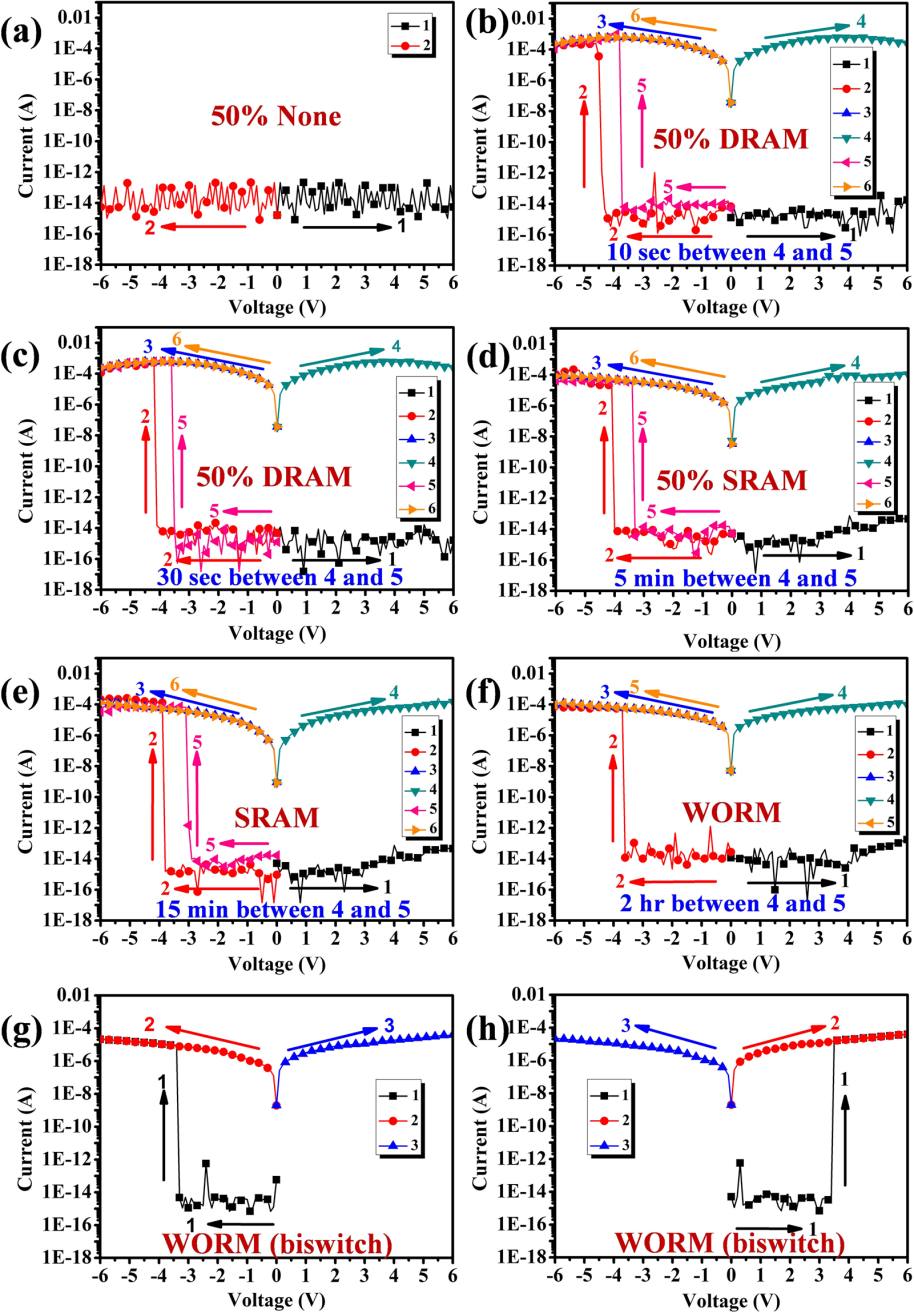
Current-voltage (I–V) features of the ITO/S-OH/TiO_2_ materials of hybrids (50 ± 3 nm)/Aluminum memory device. (**a**) and (**b**) S-OHTi5. (**c**) and (**d**) S-OHTi7. (**e**) S-OHTi10. (**f**) S-OHTi15. (**g**) and (**h**) S-OHTi30.

**Figure 6:**
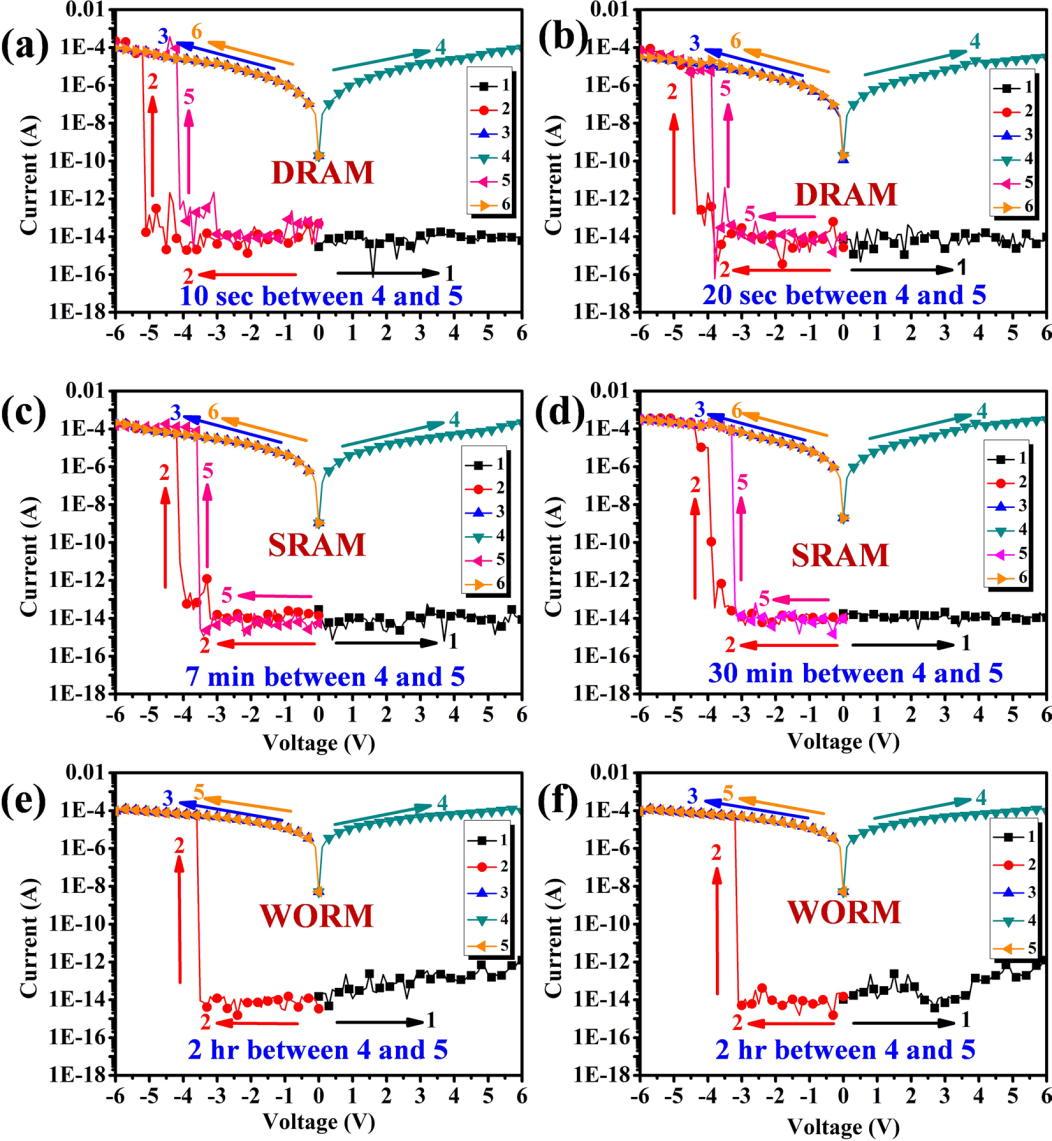
Current-voltage (I–V) features of the ITO/S-OH/ZrO_2_ materials of hybrids (50 ± 3 nm)/Aluminum memory device. (**a**) S-OHZr5 (**b**) S-OHZr7. (**c**) S-OHZr10 (**d**) S-OHZr15. (**e**) S-OHZr30. (**f**) S-OHZr50.

For the S-OH hybrid system, the I-V curves of memory devices originated from polymer hybrids with TiO_2_ and ZrO_2_ as acceptors of electron are summarized in Figs 5 and 6. I–V curves of S-OHTi5 hybrid with 5 wt % of TiO_2_ are shown in Fig. 5a and b, maintaining at OFF state with a current range 10^−13^ to 10^−15^ A in the positive sweep up to 6 V without switching to the ON state. While, a dramatic current increasing at −4.4 V during negative sweep with probability about 50% could be observed, meaning an electrical transition between OFF state and ON state (writing process). Then, the device would remain in ON state by subsequent negative (the 3^th^ sweep) and positive scans (the 4^th^ sweep). In addition, the S-OHTi5 memory device could not be reset to initial OFF state with a reverse electric field, implying non-erasable behavior. After shutting down the power for about 10 sec without an erasing process, the 5^th^ sweep was operated and demonstrated that the relaxation from ON state to the initial OFF state occurred, and the device would be switched to ON state at −3.7 V again. Hence, the device owns DRAM characteristics of rewriting capability and short retention time. The S-OHTi7 hybrid memory devices, containing 7 wt % of TiO_2_, switched from 10^−15^ to 10^−4^ A in the negative sweep at the voltage of threshold of −4.1 V, then we could read the ON state by the following negative (the 3^th^ sweep) and positive (the 4^th^ sweep) scans as shown in Fig. 5c and d, respectively. After removing the applied voltage, the ON state would return to OFF state both within 30 sec and 5 min, and they also could switch to ON state at the voltage of −3.5 V and −3.3 V again, correspondingly, denoting a volatile 50% DRAM and 50% SRAM like behaviors. In Fig. 5e, the memory device of S-OHTi10 hybrid containing 10 wt % of TiO_2_ could be switched from 10^−15^ to 10^−4^ A in the negative sweep at the voltage of threshold of −3.8 V, then could keep the ON state by the following negative (the 3^th^ sweep) and positive (the 4^th^ sweep) scans for 15 min without the applied voltage, the OFF state would be returned from the ON state in 15 min, and could switch to ON state again at threshold voltage of −3.0 V, suggesting a volatile behavior of SRAM. By further increasing TiO_2_ content to 15 wt %, the memory device based on S-OHTi15 film shown in Fig. 5f could even preserve the ON state after 2 h power-off or a longer time, indicating non-volatile write-once-read-many (WORM) memory behavior. Moreover, the device based on S-OHTi30 hybrid containing TiO_2_ up to 30 wt % could also be switched to ON state by applied a positive voltage of 3.4 V. Thus, the memory device based on S-OHTi30 film depicted in Fig. 5g and h revealed the bi-switchable characteristic caused by the smaller energy gap between the LUMO of TiO_2_ and work function of ITO shown in Fig. 4b. The stability both in ON and OFF states of WORM memory device derived from S-OHTi30 film is detailed in Figure S11 (ESI†).

Moreover, the devices of S-OH/ZrO_2_ hybrids have also been fabricated for comparison. The S-OHZr5 and S-OHZr7 hybrids memory devices containing 5 and 7 wt % ZrO_2_ behaved DRAM properties with voltage of threshold around −5.1 V (S-OHZr5) and −4.4 V (S-OHZr7) during the negative sweep as shown in Fig. 6a and b, and the retention time is only 10 sec (S-OHZr5) and 20 sec (S-OHZr7), respectively, for returning to OFF state from ON state before rewriting process.

The devices prepared from S-OHZr10 and S-OHZr15 hybrids containing 10 and 15 wt % of ZrO_2_ could be switched on at about −4.1 V (S-OHZr10) and −3.8 V (S-OHZr15) in the negative sweep and exhibited volatile behavior of SRAM with retention time about 7 min (S-OHZr10) and 30 min (S-OHZr15) shown in Fig. 6c and d, respectively. Interestingly, the S-OH/ZrO_2_ hybrid devices containing higher ZrO_2_ content of 30 wt% and 50 wt% shown in Fig. 6e and f displayed only WORM memory property without bi-switchable behavior as the results of the analogous S-OHTi30 that may be ascribed to higher LUMO energy level of ZrO_2_ than TiO_2_ without matching up with the ITO work function depicted in Fig. 4c.

The obtained polymer S-OH hybrid materials with TiO_2_ and ZrO_2_ as acceptors of electron have lower LUMO energy level that would make complex of CT stabilized and facilitated. Accordingly, in Table 3, the resulted devices of hybrid memory manifested high ON/OFF ratio (10^8^) with memory properties which are tunable from DRAM, SRAM, to WORM at various contents of titanaia and zirconia ranging from 0 wt% to 50 wt%. All the memory devices show lower threshold voltage and longer retention time with higher inorganic contents. Extraordinary, the difference of LUMO energy level between TiO_2_ and ZrO_2_ results in uniquely distinct memory behavior in terms of bi-switchable characteristic. Thus, these interesting and attractive results obtained in this study demonstrate that prepared S-OH/titania and S-OH/zirconia hybrid films with highly transparency have specific potential in the memory devices applications.

**Table 3:** Summary of S-OH, S-OH/TiO_2_, and S-OH/ZrO_2_ memory properties.

S-OH + TiO_2_/ZrO_2_	0 wt%	5 wt%	7 wt%	10 wt%	15 wt%	30 wt%	50 wt%
TiO_2_ Memory Properties	None	50% None 50% DRAM (10 sec)	50% DRAM 50% SRAM (5 min)	SRAM (15 min)	WORM	WORM (biswitch)	WORM (biswitch)
ZrO_2_ Memory Properties	None	DRAM (10 sec)	DRAM (20 sec)	SRAM (7 min)	SRAM (30 min)	WORM	WORM

## Discussion

Highly transparent and thermoplastic polyimidothioethers (PITEs) with ultra-lowest birefringence value of 0.002 have been prepared successfully by Michael polyaddition from commercially available bismaleimides and dithiol monomers in this research. The hydroxyl groups in the PITEs backbones would effectively contribute reaction sites for the bonding between organic and inorganic materials, resulting in transparent and homogeneous films of hybrid. These polymer hybrid films manifest attractive RI which is tunable (1.65–1.81 for S-OH/titania and 1.65–1.80 for S-OH/zirconia) and birefringence value lower than 0.01, which may have potential and connection to optical applications in the future. Additionally, the Abbe number and optical transparency of S-OH/ZrO_2_ hybrids are higher than those of S-OH/TiO_2_ hybrid system because of a larger band gap of ZrO_2_, demonstrating much more valuable as the optical materials. Moreover, the obtained polymer hybrid materials with titania and zirconia as acceptors of electron in S-OH system own lower LUMO energy level that would make complex of CT stabilized and facilitated. Consequently, the fabricated memory devices from these hybrid materials revealed different and tunable memory behaviors with an impressive ON/OFF ratio (10^8^) at various titania or zirconia content from 0 wt% to 50 wt%. Interestingly, the difference of LUMO energy levels between ZrO_2_ and TiO_2_ could distinctively result in various WORM type memory devices (bi-switchable WORM type for S-OHTi30, but mono-switchable WORM type for S-OHZr30).

## Materials and Methods

### Polymer synthesis

The PITEs could be easily prepared by Michael polyaddition with commercially available monomers, and the general synthetic route for preparing PITEs is shown in Fig. 1. For more details please refer to supporting information.

### Preparation of the PITE, S-OH/titania, and S-OH/zirconia hybrids films

The DMAc solution of PITE was casted onto glass substrate and removed the solvent at 80 °C for 6 h and 150 °C for 8 h under vacuum to obtain polymer films which thicknesses are about 20 μm that were used for optical, thermal measurements, and solubility tests. Figure 1 depicts the general route for preparing the hybrid films of S-OH/titania and S-OH/zirconia hybrids. For more details please refer to supporting information.

## Electronic supplementary material

0704-2017-ESI for Scientific Reports (PITE)
