# Supplementary material for: Optically Isotropic, Colorless, and Flexible PITEs/TiO2 and ZrO2 Hybrid Films with Tunable Refractive Index, Abbe Number, and Memory Properties
Source: Sci Rep. 2017 Aug 11;7:7978. doi: 10.1038/s41598-017-08544-3 (PMC5554255; doi:10.1038/s41598-017-08544-3)
Supplement: Supplementary file 1 — 0704-2017-ESI for Scientific Reports (PITE) [file 41598_2017_8544_MOESM1_ESM.pdf]

Supporting Information

Optically Isotropic, Colorless, and Flexible Polyimidothioethers/TiO_2 and ZrO_2 Hybrid Films with Tunable Refractive Index, Abbe Number, and Memory Properties

Tzu-Tien Huang,‡ Shun-Wen Cheng, ‡ Chia-Liang Tsai, and Guey-Sheng Liou*

Functional Polymeric Materials Laboratory, Institute of Polymer Science and Engineering, National Taiwan University, Taipei, Taiwan

Tel: +886-2-336-5315; E-mail: gsliou@ntu.edu.tw

List of Contents for Supplementary Material:

Materials 3

Polymer synthesis 3

Preparation of S-OH/titania and S-OH/zirconia hybrids 3

Measurements 4

Fabrication and Measurement of Memory Devices 5

Molecular Simulation 5

Fig. S1. IR spectra of **PITEs**. 6

Fig. S2. IR spectra of **S-OHTi50** and **S-OHZr50** hybrid materials. 7

Fig. S3. TMA curves of (a) **PITEs**, (b) **S-OH /TiO_2** and (c) **S-OH /ZrO_2** hybrid films with the heating rate of 10 ^{o}C/min. 8

Fig. S4. TGA traces of **PITEs**, **S-OH/TiO_2** and **S-OH/ZrO_2** hybrid materials (a), (c) and (e) in N_2, (b), (d) and (f) in air. 9

Fig. S5. TEM image and statistical data curve of S-OHTi30 materials of hybrid..........**10**

Fig. S6. TEM image and statistical data curve of S-OHZr30 materials of hybrid.. ....**11**

Fig. S7. UV-vis absorption spectra of **PITEs** films...**12**

Fig. S8. Cyclic voltammetric diagrams of the **PITEs** films on an ITO-coated glass substrate. **13**

Fig. S9. Current-voltage (I-V) characteristics of the ITO/**PITEs** (50 ± 3 nm)/Al memory device (a) **S-OH** (b) **CH_2-OH** and (c) **SO_2-OH**....................................**14**

Fig. S10. Calculated molecular orbitals and respective energy levels of the basic units for PITEs (a) S-OH (b) CH_2-OH and (c) SO_2-OH..**15**

Fig. S11. The stability of memory devices at the ON and OFF states of the ITO/**S-OH** hybrid materials (50 ± 3nm)/Al devices (a) **S-OHTi30** and (b) **S-OH Zr30**. **16**

Table S1. Inherent viscosity, GPC data and solubility behavior of PITEs and references..**17**

Table S2. Redox potential and energy level of PITEs **18**

Experimental

Materials

4,4'-(Diaminodiphenylsulfide)bismaleimide (S-BMI)[1] (mp: 187°C) and 4,4'-(diaminodiphenylsulfone)bismaleimide (SO_2-BMI)[2] (mp: 252 °C) were readily synthesized by reacting the respective diamines with maleic anhydride according to the reported procedure. Commercially available monomers such as 4,4'-(diaminodiphenylmethane)bismaleimide (CH_2-BMI), and (2*S*,3*S*)-1,4-disulfanyl-butane-2,3-diol (DT-OH), *N*,*N*-dimethylacetamide (DMAc) (TEDIA), and triethyl-amine (TEA) (Across) and other reagents were used as received from commercial sources.

Polymer synthesis

The synthesis of PITE S-OH was used as an example to illustrate the general synthetic route used to produce the PITEs. The mixture of bismaleimide S–BMI (0.753 g, 2.0 mmol), 5mL of m-cresol, and DT–OH (0.309 ml, 2 mmol) was prepared and stirred for 10 min. Then, 10 μL of triethylamine was added slowly into the mixture, and the polymerization proceeded at room temperature for 4 h. The obtained polymer solution was poured slowly into 300 mL methanol giving rise to a white fiber-like precipitate and dried under reduced pressure at 100 °C for 6 hours.

Preparation of S-OH/titania and S-OH/zirconia hybrids

The synthesis of S-OHTi50 was used as an example to illustrate the general route for producing the hybrid films of S-OH/titania (S-OHTiX). Firstly, 0.05 g of S-OH was dissolved in 3 mL of DMAc, 0.1mL of HCl was added very slowly into the S-OH solution, and stirred at room temperature for 30 min. Then, 0.152 mL (0.45 mmole) of $Ti(OBu)_4$ dissolved in 0.152 mL of butanol was added drop-wisely into the above solution by a syringe, and then stirred at room temperature for 2 hours. Finally, the resulting precursor solution was filtered through a 0.45 mm PTFE filter and drop-coated onto glass substrates. The preparation of S-OH/zirconia (S-OHZrX) hybrid is similar to the S-OHTiX mentioned above, and the S-OHZr50 was used as an example to illustrate the procedure of the hybrid S-OHZrX. Firstly, 0.05 g of S-OH was dissolved in 3mL of DMAc, and then 0.10 mL of acetic acid was added very slowly into the polymer solution and further stirred at room temperature for 30 min. Then, 0.21 mL (0.62 mmole) of $Zr(OBu)_4$ dissolved in 0.21 mL of butanol was added drop-wisely into the above solution by a syringe, and then stirred at room temperature for 10 min. Finally, the resulting precursor solution of was filtered through a 0.45 mm PTFE filter and drop-coated onto glass substrates. For the preparation of optical hybrid films with thickness about 20±3 μm, the above precursor solution was cast

onto glass plate followed by a subsequent heating program at 80 ^{o}C for 6 h, and 150 ^{o}C for 8 h under vacuum. In addition, the above prepared solution was also spin-coated onto a glass plate or silicon wafer at 1000–2500 rpm for 1 min. The obtained film was then treated by the heating process of 80 ^{o}C for 30 min, and 150 ^{o}C for 60 min, respectively, to afford hybrid thin film with thicknesses of 500-600 nm. Then, these resulting S-OH hybrid thick and thin films were further treated via a hydrothermal process by placing them into the water vapor at 100 ^{o}C for 12 h. The flexible and transparent S-OHMX hybrid optical films with different titania or zirconia contents could be successfully prepared.

Measurements

Fourier transform infrared (FT-IR) spectra were recorded on a PerkinElmer Spectrum 100 Model FT-IR spectrometer with resolution 1 cm^{-1} and number of scans 4. The inherent viscosities were determined at 0.5 g/dL concentration using Tamson TV-2000 viscometer at 30 ^{o}C. Thermogravimetric analysis (TGA) conducted with a TA Instruments Q50, and experiments were carried out on approximately 3-5 mg film samples heated in flowing nitrogen or air (flow rate = 20 cm^3/min) at a heating rate of 20 ^{o}C/min. Coefficient of thermal expansion (CTE) and glass transition temperatures (T_g) were measured by a dilatometer (TA instrument SI-5 TMA Q400EM). The TMA experiments were conducted from 40 to 300 ^{o}C at a scan rate of 10 ^{o}C/min by a tensile probe under an applied constant load of 50 mN. T_g was taken as the onset temperature of probe displacement on the TMA traces, and the CTE data were determined in the range of 50–150 ^{o}C. Ultraviolet-visible (UV-vis) spectra of the obtained films were recorded on Hitachi U-4100 UV-vis-NIR spectrophotometer. An ellipsometer (SOPRA, GES-5E) was used to measure the refractive index (n) of the prepared films in the wavelength range of 300–800 nm, and the thickness (h) was also determined simultaneously. In-plane (n_{TE}), and out-of plane (n_{TM}) refractive indices of the films formed on the silica substrates were measured using a prism coupler (Metricon, PC-2000) at wavelengths of 632.8 nm at room temperature. The in-plane/out-of-plane birefringence (Δn) was calculated as $\Delta n = n_{TE} - n_{TM}$. The nanostructure of the prepared polymer hybrid films was examined by using a JOEL JEM-1230 transmission electron microscope (TEM) at an operating voltage of 100 kV. Cyclic voltammetry (CV) was performed with a Bioanalytical System Model CV-27 and conducted with the use of a three-electrode cell in which ITO (polymer films area about 0.5 * 1.2 cm^2) was used as the working electrode and a platinum wire as the auxiliary electrode at a scan rate of 100 mV s^{-1} against a Ag/AgCl reference electrode in anhydrous CH_3CN, using 0.1M of TBAP as the supporting electrolyte. All cell potentials were taken by using a homemade Ag/AgCl, KCl (sat.) reference electrode.

Fabrication and measurement of the memory devices

The memory devices were fabricated with the configuration of ITO/thin film/Al. The ITO glass used for memory devices was cleaned by ultra-sonication with water, acetone, and isopropanol each for 30 min. The hybrid thin films were prepared according to the previous procedure using ITO as a substrate, and the film thickness was adjusted to be around 50 nm. Finally, a 300 nm thick Al top electrode was thermally evaporated through the shadow mask (recorded device units of 0.5 * 0.5 mm^2 in size) at pressure of 10^{-7} Torr with a depositing rate of 3–5 Å s^{-1}. The electrical characterization of the memory device was performed using a Keithley 4200-SCS semiconductor parameter analyzer equipped with a Keithely 4205-PG2 arbitrary waveform pulse generator. ITO was used as the cathode (maintained as common), and Al was set as the anode during the voltage sweep. The probe tip used 10 mm diameter tungsten wire attached to a tinned copper shaft with a point radius <0.1 mm (GGB Industries, Inc.).

Molecular simulation

Molecular simulation in this study was carried out with the Gaussian 09 program package. Equilibrium ground state geometry and electronic properties of basic unit in the polyimide were optimized by means of the density functional theory (DFT) method at the B3LYP level of theory (Beckesstyle three-parameter density functional theory using the Lee–Yang–Parr correlation functional) with the 6-31G(d) basic set.

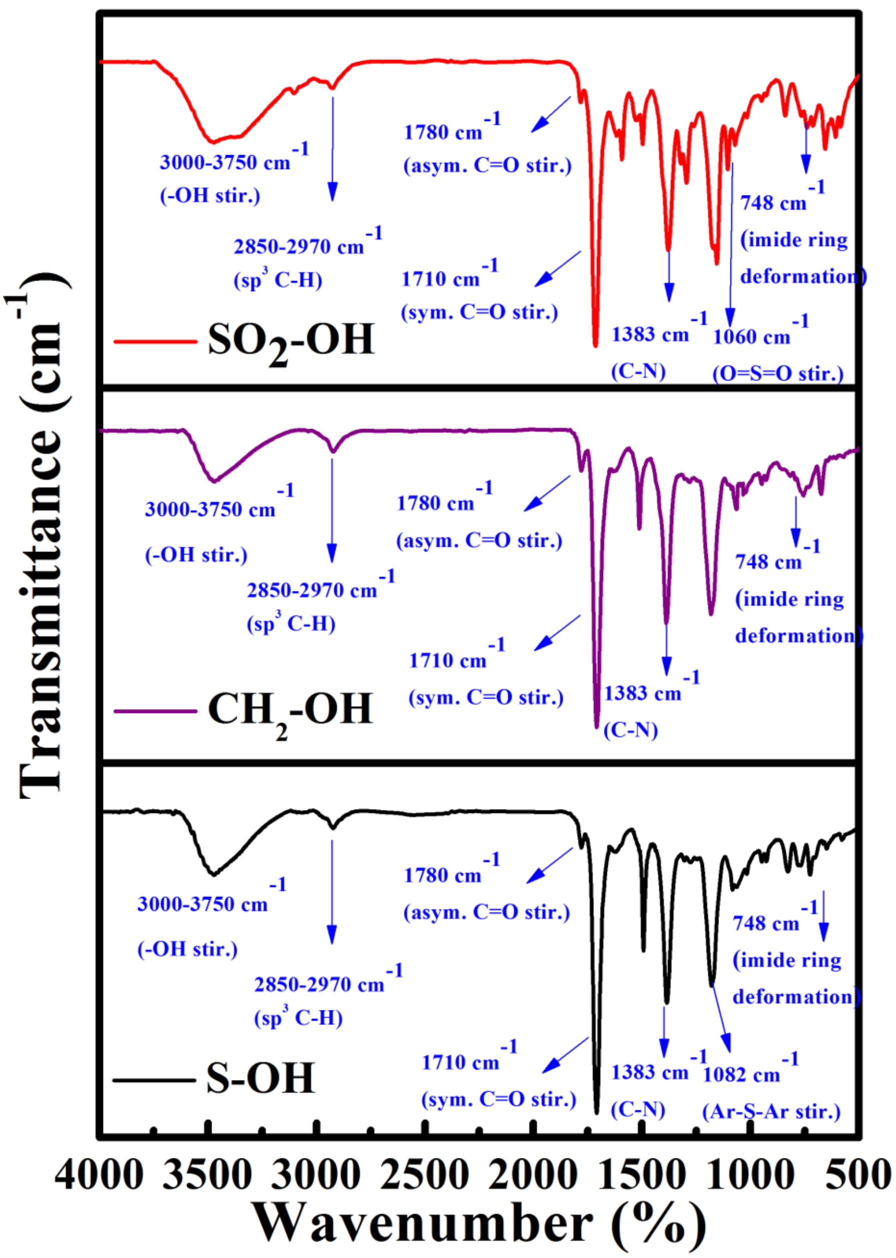

Fig. S1. IR spectra of **PITEs**.

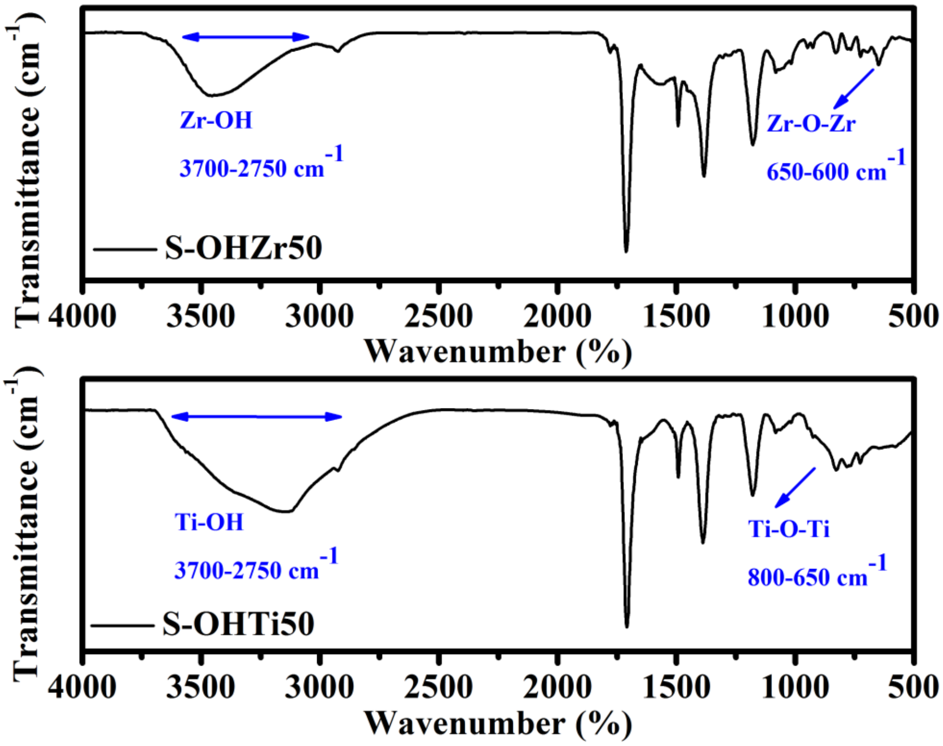

Fig. S2. IR spectra of **S-OHTi50** and **S-OHZr50** hybrid materials.

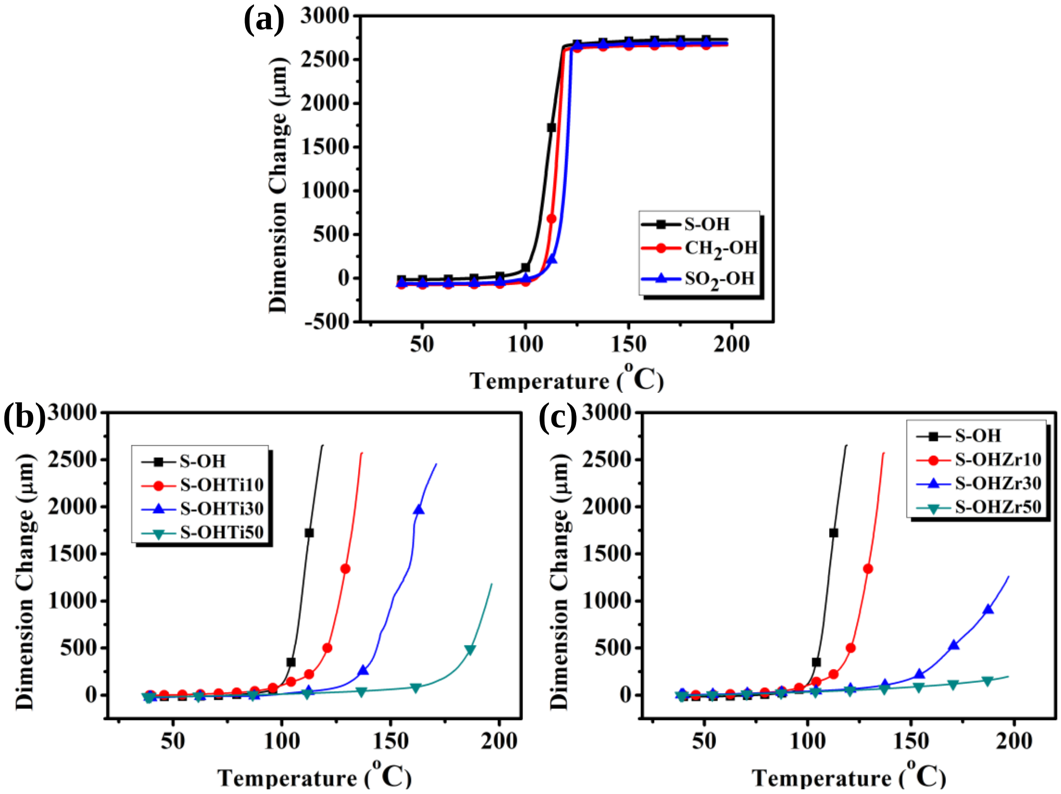

Fig. S3. TMA curves of (a) **PITEs**, (b) **S-OH/TiO_2** and (c) **S-OH/ZrO_2** hybrid films with the heating rate of 10 ℃/min.

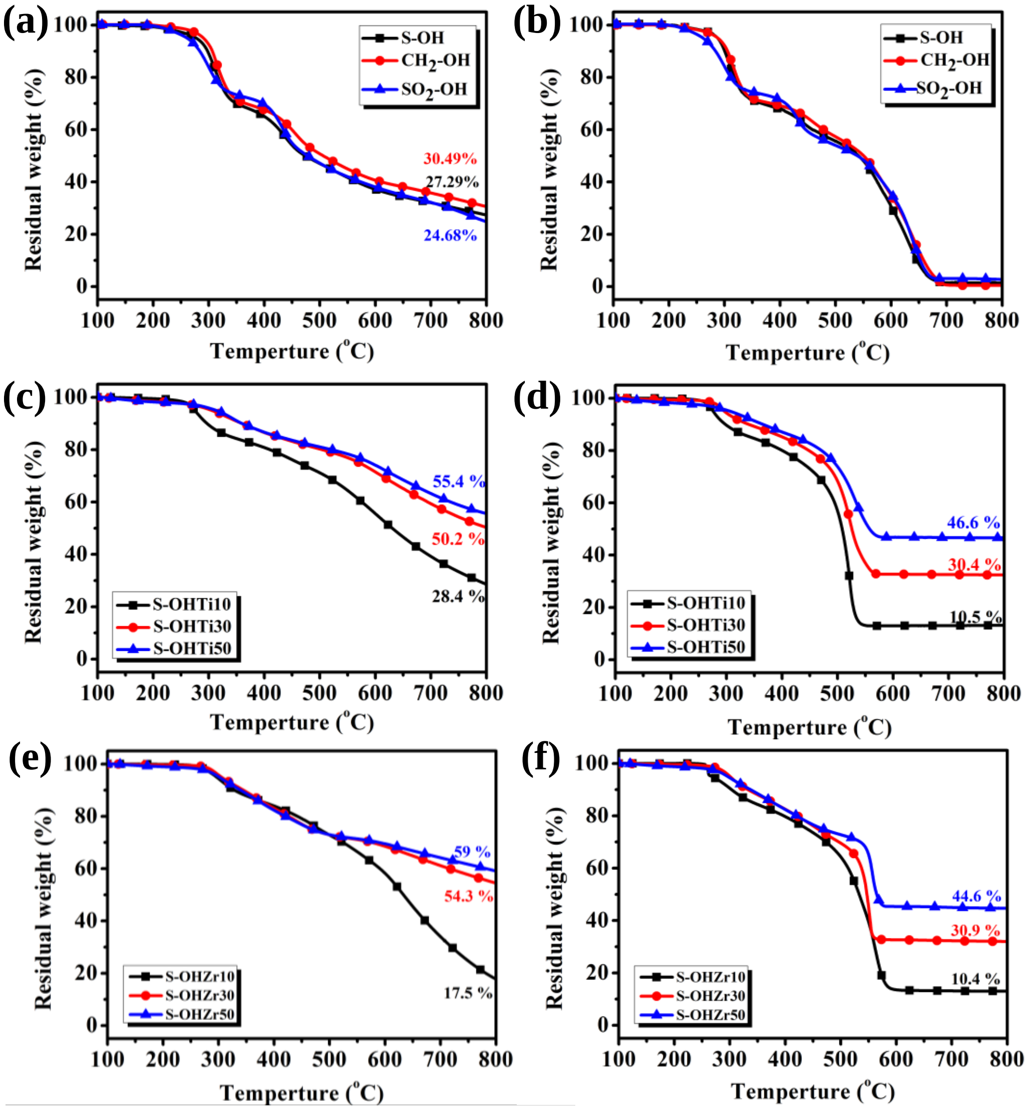

Fig. S4. TGA traces of **PITEs**, **S-OH/TiO2** and **S-OH/ZrO2** hybrid materials (a), (c) and (e) in N_2, (b), (d) and (f) in air.

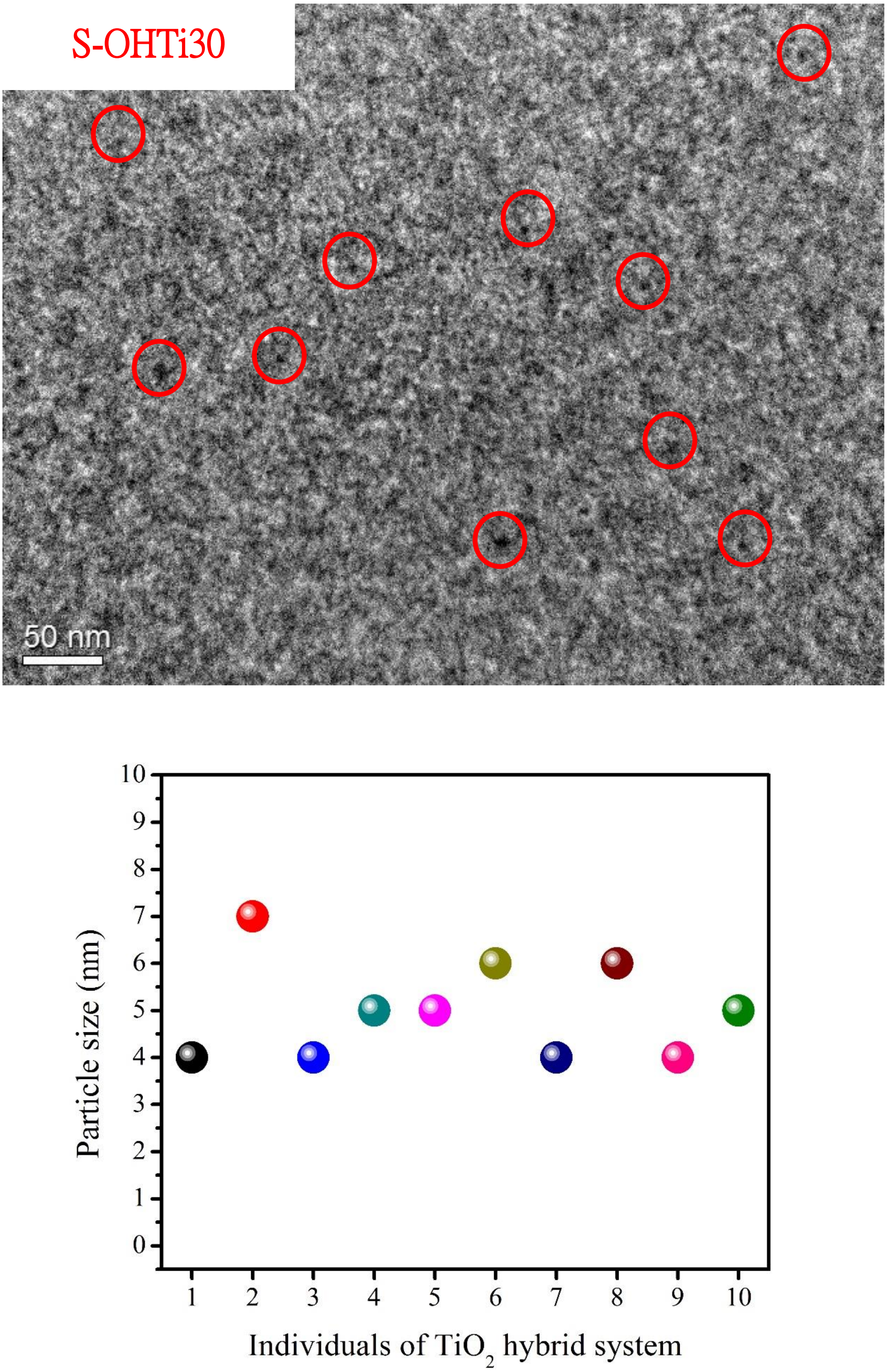

Fig. S5. TEM image and statistical data curve of S-OHTi30 materials of hybrid.

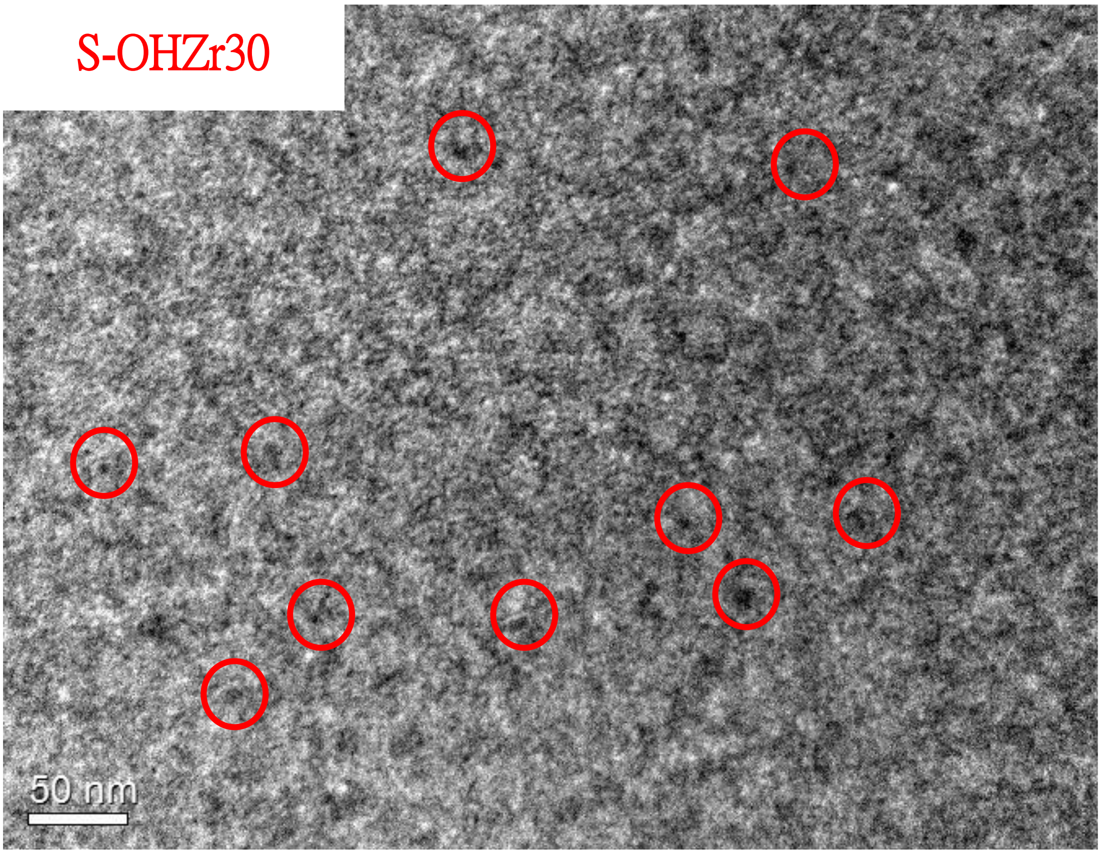

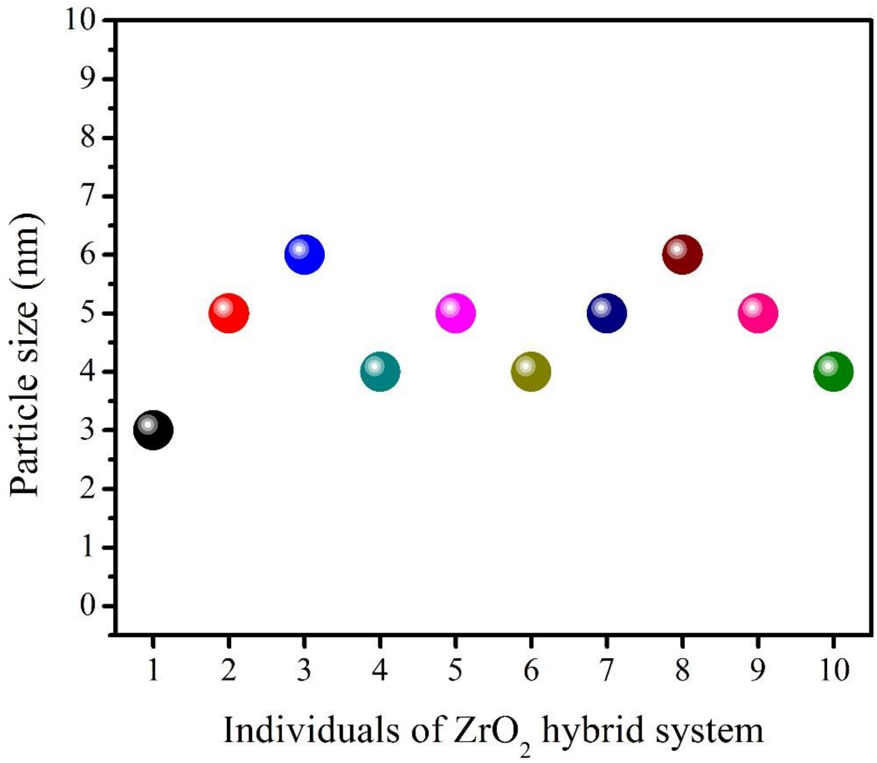

Fig. S6. TEM image and statistical data curve of S-OHZr30 materials of hybrid.

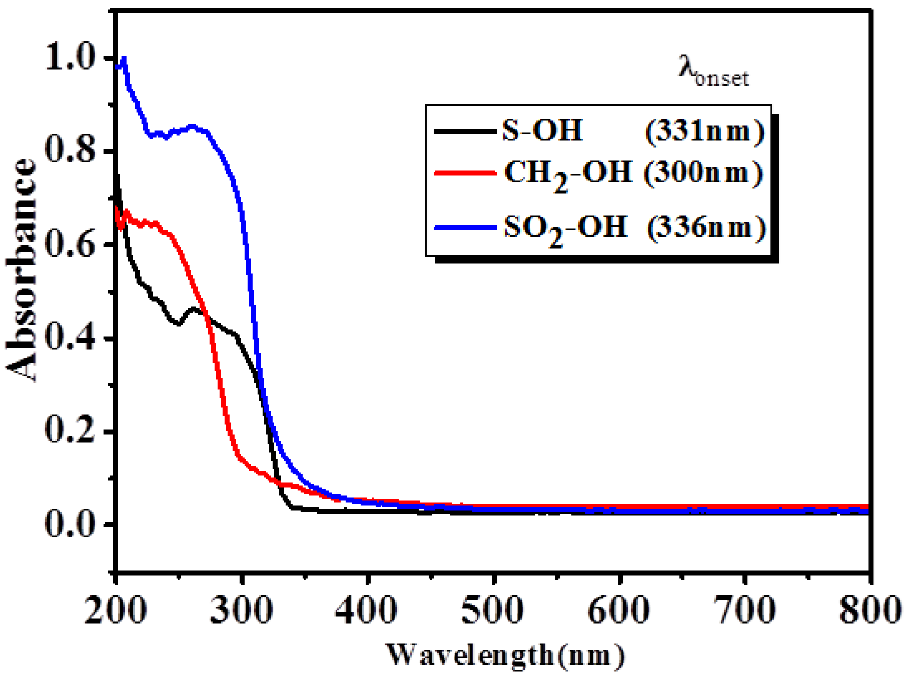

Fig. S7. UV-vis absorption spectra of **PITEs** films.

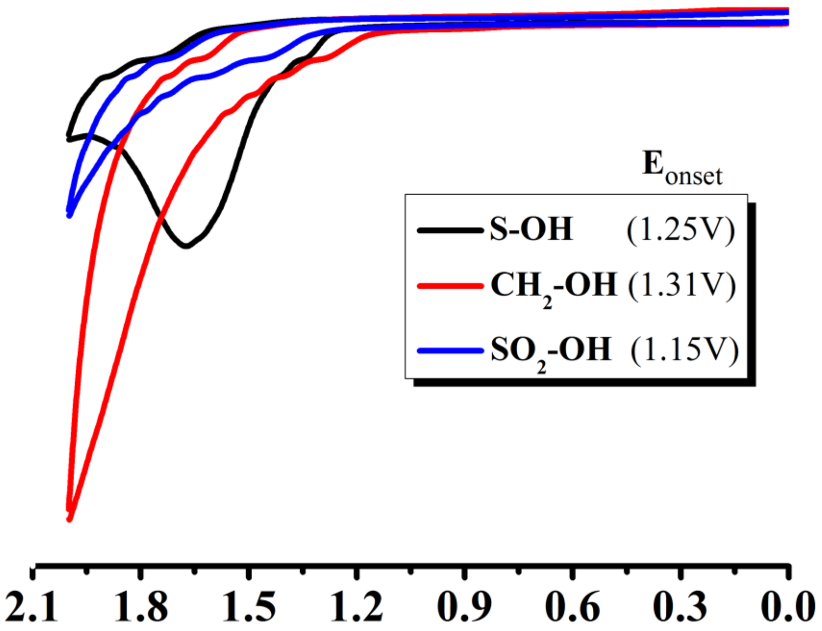

Fig. S8. Cyclic voltammetric diagrams of the **PITEs** films on an ITO-coated glass substrate.

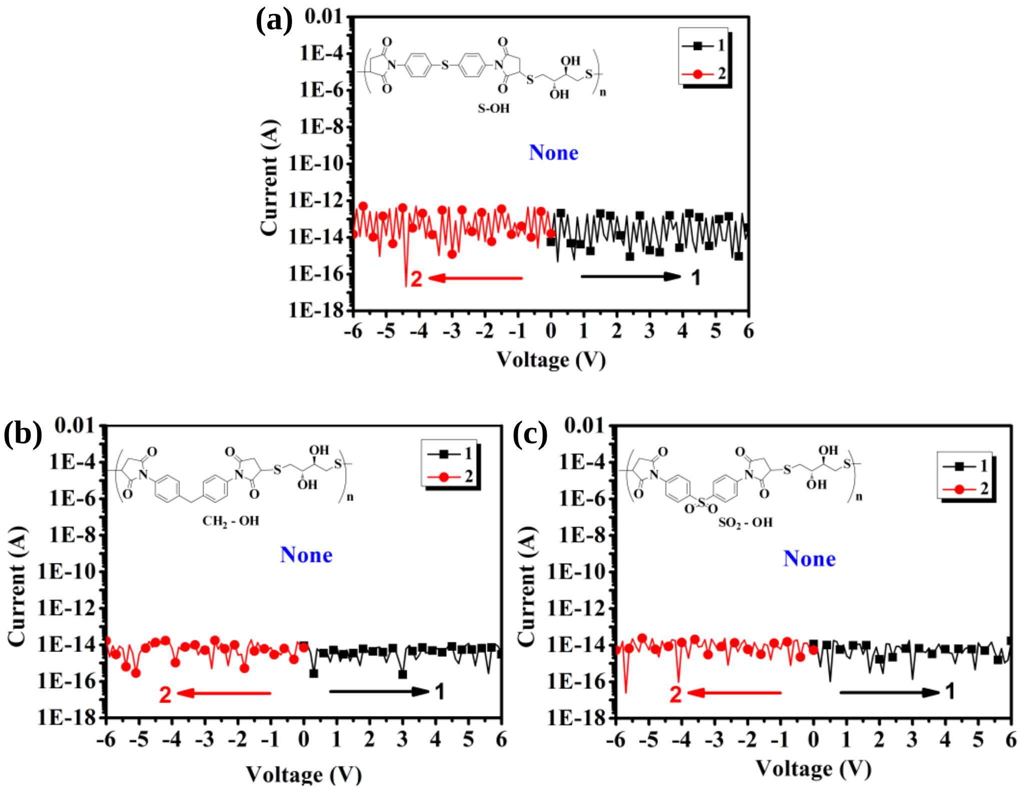

Fig. S9. Current-voltage (I-V) characteristics of the ITO/**PITEs** (50 ± 3 nm)/Al memory device (a) **S-OH** (b) **CH_2-OH** and (c) **SO_2-OH**.

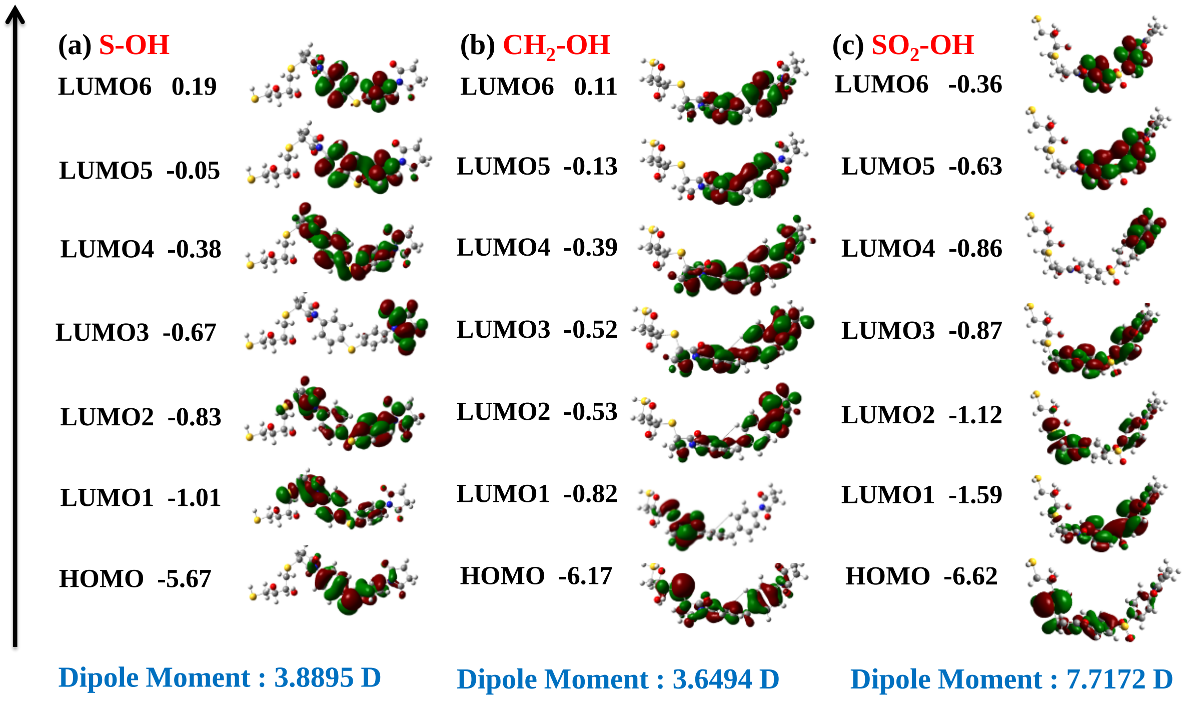

Fig. S10. Calculated molecular orbitals and respective energy levels of the basic units for PITEs (a) S-OH (b) CH_2-OH and (c) SO_2-OH.

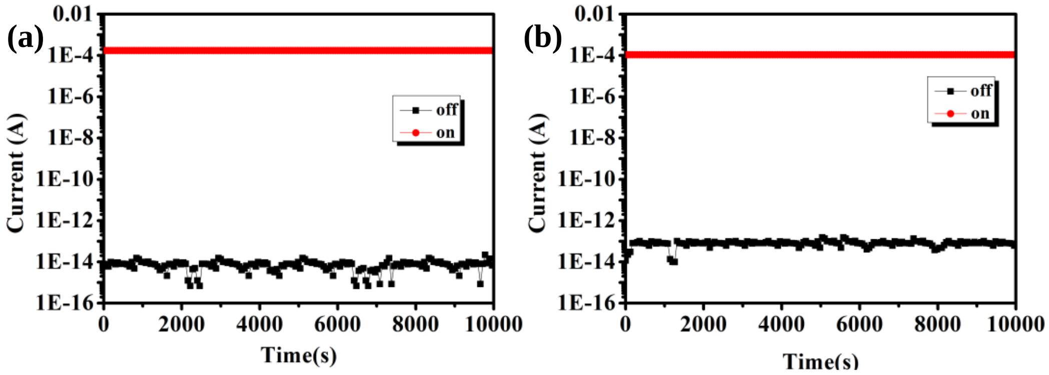

Fig. S11. The stability of memory devices at the ON and OFF states of the ITO/S-OH hybrid materials (50 ± 3nm)/Al devices (a) **S-OHTi30** and (b) **S-OH Zr30**.

Table S1. Inherent viscosity, GPC data and solubility behavior of **PITEs.**

Polymer	η^a (dL/g)	GPC Data[b]			Solubility in various solvents[d]						
		M_n	M_w	PDI[c]	NMP	DMAc	DMF	DMSO	*m*-Cresol	THF	$CHCl_3$
**S-OH**	1.01	60500	132100	2.18	++	++	++	+-	++	-	-
**CH_2-OH**	1.25	59400	133700	2.25	++	++	++	+-	++	-	-
**SO_2-OH**	0.54	55200	110970	2.01	++	++	++	+-	++	-	-

1. B. S. Rao, R. Sireesha and A. R. Pasala, *Polym. Int*., 2005**, 54**, 1103.
2. J. G. Liu and M. Ueda, *J. Mater. Chem.*, 2009**, 19**, 8907.

Table S2. Redox potential and energy level of PITEs.

Polymer	UV-vis absorption (nm)	Oxidation potential (V)[a]	E_g [b] (eV)	HOMO[c] (eV)	LUMO (eV)
	λ_{onset}	E_{onset}			
S-OH	331	1.25	3.74	-5.69	-1.95
CH_2-OH	300	1.15	4.13	-5.59	-1.46
SO_2-OH	336	1.31	3.69	-5.75	-2.06

[a] The data compared with Ag/AgCl in CH_3CN.
[b] The data were calculated by the equation: $Eg=1240/\lambda_{onset}$.
[c] The HOMOs were calculated from CV referenced to ferrocene.
